# CSGNN: Contamination Warning and Control of Food Quality via Contrastive Self-Supervised Learning-Based Graph Neural Network

**DOI:** 10.3390/foods12051048

**Published:** 2023-03-01

**Authors:** Junyi Yan, Hongyi Li, Enguang Zuo, Tianle Li, Chen Chen, Cheng Chen, Xiaoyi Lv

**Affiliations:** 1 College of Software, Xinjiang University, Urumqi 830046, China; 2 Guangzhou Panyu Polytechnic, No. 1342 Shiliang Road, Guangzhou 511483, China; 3 College of Information Science and Engineering, Xinjiang University, Urumqi 830046, China

**Keywords:** food quality safety, contamination warning, contrastive learning, self-supervised learning, graph neural networks

## Abstract

Effective contamination warning and control of food quality can significantly reduce the likelihood of food quality safety incidents. Existing food contamination warning models for food quality rely on supervised learning, do not model the complex feature associations between detection samples, and do not consider the unevenness of detection data categories. In this paper, To overcome these limitations, we propose a Contrastive Self-supervised learning-based Graph Neural Network framework (CSGNN) for contamination warning of food quality. Specifically, we structure the graph for detecting correlations between samples and then define the positive and negative instance pairs for contrastive learning based on attribute networks. Further, we use a self-supervised approach to capture the complex relationships between detection samples. Finally, we assessed each sample’s contamination level based on the absolute value of the subtraction of the prediction scores from multiple rounds of positive and negative instances obtained by the CSGNN. Moreover, we conducted a sample study on a batch of dairy product detection data in a Chinese province. The experimental results show that CSGNN outperforms other baseline models in contamination assessment of food quality, with AUC and recall of unqualified samples reaching 0.9188 and 1.0000, respectively. Meanwhile, our framework provides interpretable contamination classification for food detection. This study provides an efficient early warning method with precise and hierarchical contamination classification for contamination warning of food quality work.

## 1. Introduction

Food safety issues are of increasing concern to international organizations and people worldwide. Efficient surveillance and early warning programs can effectively reduce the probability of food safety accidents. Currently, many international organizations and countries have established monitoring systems to ensure food quality safety [1,2,3,4]. Similarly, China has gradually improved its national food safety risk assessment system [5,6]. For example, in 2009, China enacted the Food Safety Law of the People’s Republic of China. In 2011, the China National Center for Food Safety Risk Assessment (CFSA) was established. In 2018, the revised version of the Food Safety Law of the People’s Republic of China included food safety assessment as a scientific basis for implementing regulations and setting standards. Therefore, developing food safety risk assessment methods can help systematize and standardize China’s food safety regulatory system. Contamination of food quality is one of the significant causes of food safety risks. Contamination warning and control of food quality are closely related to food safety risk assessment and are an essential part of food safety regulations.

The existing mainstream contamination warning methods mainly include hierarchical relationship analysis-based methods [7,8,9], Bayesian network-based methods [10,11], and artificial neural network (ANN)-based methods [12]. However, these approaches exhibit the following deficiencies.

**Figure 1:**
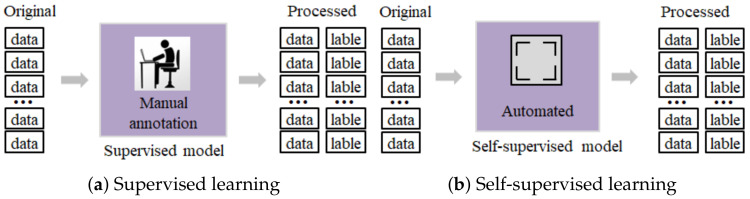
Supervised learning versus self-supervised learning.

1. They rely on supervised learning [13]. Still, manual labeling of detection data labels will significantly increase the time cost and require operators to have an explicit knowledge of data category classification. Once a casual error in classifying data categories occurs, it will lead to a series of subsequent tasks with persistent interference from subjective factors, which is fatal in practical application scenarios. The supervised learning process on raw data is shown in (a) in Figure 1.
2. They use balanced training data or do not consider the category imbalance in the training data. Data category imbalance is a significant quantitative difference in the sample size of different labels in the data, which is common in practical scenarios. Category imbalance can limit the model’s performance to varying degrees [14,15], so it is critical to investigate how to adopt strategies to address the data category imbalance while ensuring relatively good performance [16].
3. They do not adequately capture topological information between detection samples. The data obtained in the detection process has the characteristics of complexity, nonlinearity and discreteness, which means that we need to pay attention to the detection data’s attribute information and topology information as much as possible to realize the contamination warning of food quality more accurately.

**Figure 2:**
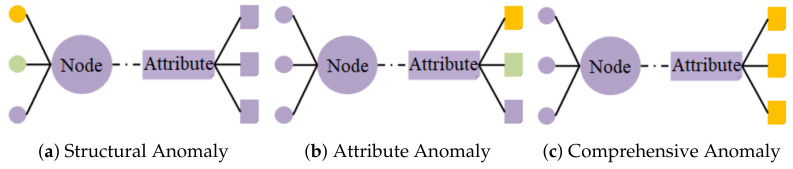
The attribute network catches three exceptions. Subgraph (**a**) belongs to the structural exception. That is, there are connection nodes that do not match all properties. Subgraph (**b**) belongs to the attribute exception. That is, some attributes do not match all nodes. Subgraph (**c**) belongs to the comprehensive exception. That is, there are both structural exceptions and attribute exceptions.

Contrastive learning is a promising solution to the above limitations. Contrastive learning uses a self-supervised approach to construct supervised information from the data, essentially addressing the reliance on manual labeling [17], processed as in (b) in Figure 1. Contrastive learning focuses on learning common features between instances of the same class and distinguishing differences between different classes by modeling the relationship between each node and some of its adjacent substructures [18]. Moreover, contrastive learning has powerful advantages in graph representation learning [19,20], especially for anomaly detection in attribute networks [21]. The learned embeddings in attribute networks include attribute and structure information, effectively capturing topological and attribute information in the network [22,23,24]. Figure 2 shows three anomalies that attribute networks are committed to capturing. The contamination warning of food quality task aims to mine all quality-contaminated unqualified samples and potentially contaminated qualified samples, that is, to discover anomalous samples whose characteristic information differs from most normal samples, which is similar to the principle of anomaly detection in attribute networks [25]. Graph neural networks (GNNs) model complex correlations among sample individuals. Therefore, attribute network-based contrastive learning has the potential to be applied to the contamination warning of food quality tasks.

Based on the above observations, we propose a novel Contrastive Self-supervised learning-based Graph Neural Network framework (CSGNN for abbreviation) for contamination warning and control of food quality. Specifically, we first construct the food detection data into an attribute graph containing attributes and structural information [26,27]. Further, the self-supervised contrastive learning module is trained by sampling the positive and negative instance pairs obtained from the complete attribute graph. Therefore, CSGNN can efficiently exploit detection data’s structural information and contrastive association information to accomplish contamination warning and control of food quality. In brief, the main contributions of the food safety contamination early warning framework proposed in this paper are as follows.

1. An end-to-end contamination warning and control framework for food quality is proposed by us, which can efficiently mine quality-contaminated unqualified samples and potentially contaminated qualified samples in food detection data by obtaining contamination values from food detection data.
2. A contrastive self-supervised learning scheme for contamination warning and control of food quality is proposed by us. Contrastive learning solves the dependence of the previous method on the balance of data categories. Meanwhile, self-supervised learning efficiently solves the problems of being easily interfered with by subjective factors and high time costs in the manual labeling operation of data categories in practical applications.
3. GNNs are used for information transfer. The CSGNN considers the data nodes’ attributes and structural information by constructing an attribute graph. To the best of our knowledge, it is also the first time graph algorithms have been applied to food safety risk assessment-related tasks.
4. The data in the actual scenarios verify that the contamination warning effect of the proposed algorithm is better than that of the current mainstream model. On a batch of dairy product detection data in a specific province in China, we compared the CSGNN framework with the mainstream model. Under self-supervision, the recall of unqualified samples of CSGNN reached 1.0000, which was more than 13% higher than the sub-optimal model. In addition, CSGNN completed the contamination classification of the food detection data based on the contamination value of each sample.

## 2. Related Work

### 2.1. Contamination Assessment Models for Food Quality

There are many types of traditional contamination assessment models for food quality. Various assessment methods exhibit different performances. Specifically, back-propagation (BP) may fall into an optimal local solution and lead to training failure during the training process [28]. The support vector machine (SVM) can guarantee to find the optimal global resolution with the help of convex optimization, which has been applied in various detection tasks [29,30]. Still, it cannot fully exploit the potential risks of food safety data. Bayesian network models have also been applied to contamination assessment tasks for food quality [31,32]. Still, they usually require modeling with expert knowledge, resulting in model performance limited by specialist experience.

With the development of deep learning, deep neural network models (DNNs) have shown promising potential in mining data features [33,34], which provides new ideas for contamination assessment for food quality. Nogales et al. applied multilayer perceptron (MLP) and one-dimensional convolutional neural network (Conv1D) to food quality contamination prediction workflow for the RASFF data launched by European Union, combining entity embedding to obtain better prediction accuracy than machine learning models [35]. Geng et al. used deep radial basis function (DRBF) neural networks combined with hierarchical analysis (AHP) to assess the contamination of food detection data [8], which enhanced the data representation of RBF shallow networks while avoiding getting trapped in local optima. However, these models are highly influenced by subjective metrics and cannot adequately capture the correlation information among complex detection data. In contrast, this paper proposes for the first time to use GNN to model the samples directly and the association information between them [36] to mine the association information in the detection data, ultimately, to achieve a more precise contamination assessment for food quality.

### 2.2. Contrastive Learning

Contrastive learning is an essential part of self-supervised learning. It completes the representation of data features by constructing a pair of instances and feeding them into the contrastive learning module. Contrastive objects and contrastive losses highlight inconsistencies between the different classes and similarity features between the same classes, respectively, which align with the original goals of downstream tasks such as classification and detection [37].

With the development of GNN, contrastive learning has also been applied to the training of GNN. DGI captures the global structural information in the network by maximizing the interaction information between the local and global input [19]. GraphCL learns node embeddings by maximizing the representational similarity between intrinsic features and link structures for local subgraphs of the same node [38]. Through a contrastive loss, SUBLIME maximized the mutual information between anchor maps and learned structure maps [39]. Although contrastive self-supervised learning has improved the performance of GNNs in speech recognition and visual representation learning [40,41], to the best of our knowledge, the current study is the first to propose its use as a contamination assessment model for food quality. Due to the particularity of the contamination assessment task, we designed the contrastive learning module to focus on the local information of the data rather than the global information, which will help the model to mine the data features of contaminated samples more efficiently.

## 3. Materials and Methods

In this paper, we use bold lowercase letters (e.g., X), bold uppercase letters (e.g., X), and fancy letters (e.g., G) to denote vectors, matrices, and sets, respectively. The main symbols used in this paper are summarized in Table 1.

### 3.1. Problem Definition and Data Source

#### 3.1.1. Problem Definition

Given a set of food detection data with *N* samples and *V* testing indicators $x_{v1,\ldots ,}x_{vn}$, first construct an attribute graph G=(V,E,X) for the detection data, where V is the set of nodes of G (|V|=N=n), E is the set of edges of G, and $X\in R^{n\times d}$ is the attribute matrix of G (d=V). The objective is to compute the contamination value $f\left(s_{i}\right)$ for each sampled sample $s_{i}$ (a higher contamination value means higher hazardous contamination of the sample presence). By ranking the contamination values of all samples, the model is based on the lowest contamination value *W* of the unqualified samples and the more obvious boundary value *U* between the contamination values of the contaminated samples and the negligible contaminated samples to achieve the contamination ranking of the detection data.

#### 3.1.2. Data Source

In this paper, 2158 detection data of sterilized dairy products(qualified samples: unqualified samples = 2117:41) from 2013 to 2021 provided by the Institute of Product Quality Supervision and Inspection in a Chinese province, were used to test the contamination assessment capability for food quality of the models. According to the National Food Safety Standard of China [42], the testing indicators of sterilized dairy products include five categories: sensory indicators, physical and chemical indicators, contaminant index, mycotoxin index and microbial index. The selection of contamination assessment for food quality testing indicators should be considered comprehensively in terms of operability and validity. According to the obtained detection data, we aim to scientifically select the testing indicators corresponding to the factors that may cause food quality contamination. Since the microbial index in the detection data has met the requirements [8], we selected the physicochemical index and mycotoxin index from the testing indicators of sterilized milk specified in the national standards as the evaluation criteria for food quality contamination, where the physicochemical index includes lactose, nonfat milk solids, protein, acidity and fat for a total of five indicators, In addition, the mycotoxin index refers to aflatoxin M${}_{1}$. Table 2 shows the six testing indicators’ specific requirements and testing methods. And Table 3 presents detection data for partially sterilized dairy products.

### 3.2. Contamination Warning of Food Quality Based on Contrastive Self-Supervised Learning

**Figure 3:**
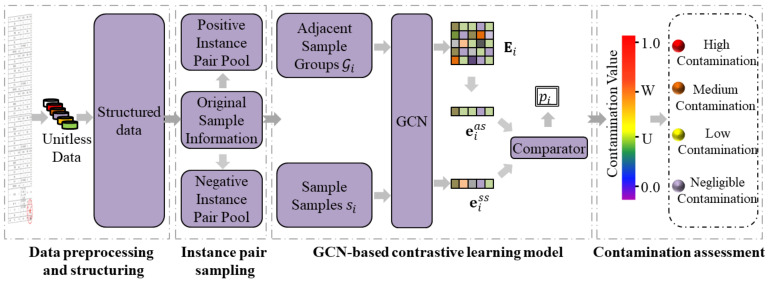
The overall framework of CSGNN. First, the framework preprocesses and structures the raw detection data to better mine the complex relationships in the detection data. Then, the local subgraph sampling strategy is used to obtain positive and negative instance pairs, which are trained by the GCN-based contrastive learning model and get the contamination value of each sample. Finally, the contamination assessment for food quality is carried out by the contamination value of the sample.

In this section, we describe the overall framework of CSGNN, as shown in Figure 3. The CSGNN framework consists of four parts: data preprocessing and structuring, contrastive instance pair sampling, GCN-based contrastive learning, and contamination assessment. First, the raw data are preprocessed and structured better to implement feature mining for detection data with complex correlations. Next, the local subgraph sampling strategy of RWR is used to obtain positive and negative instance pairs. After that, we use the GCN-based contrastive learning model to train each batch of instance pairs. Finally, all the detection samples will be randomly traversed as sampling samples. The contamination assessment of the detection samples is completed by evaluating the consistency between the sampling samples and the adjacent sample groups.

The food quality hazard contamination considered in this paper refers to the contamination of food quality due to testing indicators that do not meet the standard requirements. CSGNN is based on contrastive learning to measure the consistency between the performance of the sampled samples and their adjacent sample groups in each testing indicator. When the samples show a higher degree of inconsistency with the adjacent sample groups, their contamination values are higher, and the possibility of quality hazard contamination is more significant.

#### 3.2.1. Data Preprocessing and Structuring

**Figure 4:**
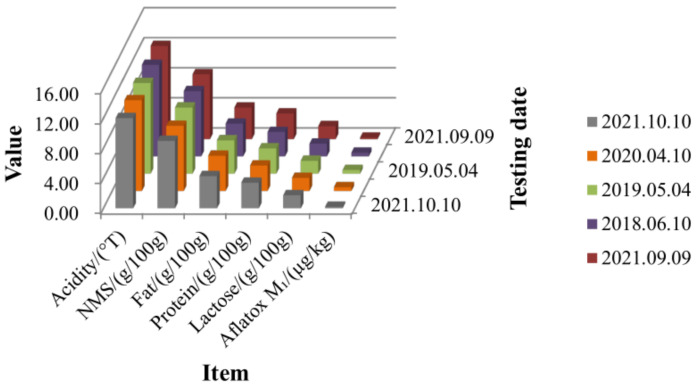
Visualization of raw partial detection data values for some sterilized dairy products.

We visualized part of the raw detection data for sterilized dairy products in Table 3, as shown in Figure 4. It can be seen from the figure that there are significant dimensional differences between the different testing indicators.

As is demonstrated in the first part of Figure 3, we used the minimum-maximum normalization method to transform the raw data into unitless data to eliminate the dimensional differences among different testing indicators. According to the different requirements of the food safety standards for the six testing indicators, we classified them into three categories: positive indicators, inverse indicators, and oscillatory indicators, as shown in Table 4. The positive indicator is one whose hazardous contamination increases as the indicator’s value increases. Conversely, the inverse indicator is one whose hazardous contamination decreases as the indicator’s value increases. In addition, the closer the oscillator is to the specified interval, the less contamination there is, and the further it is from that interval, the more contamination there is. Equations (1)–(3) normalize the three categories of indicators, respectively. Ultimately, the greater the normalized data value, the greater its hazardous contamination.

(1) $$x_{vn}^{p}=\frac{x_{vn}-x_{v}^{\min}}{x_{v}^{\max}-x_{v}^{\min}}$$

(2) $$x_{vn}^{r}=1-\frac{x_{vn}-x_{v}^{\min}}{x_{v}^{\max}-x_{v}^{\min}}=\frac{x_{v}^{\max}-x_{vn}}{x_{v}^{\max}-x_{v}^{\min}}$$

(3) $$x_{vn}^{o}=\frac{\left|x_{vn}-x_{v}^{\mathrm{mean}}\right|}{x_{v}^{max}-x_{v}^{\min}}$$

where, $x_{v}^{\max}=\max\left\{x_{v1},x_{v2},\ldots ,x_{vn}\right\}$, $x_{v}^{\min}=\min\left\{x_{v1},x_{v2},\ldots ,x_{vn}\right\}$, $x_{v}^{\mathrm{mean}\;}=\frac{\sum_{i=1}^{n}x_{vi}}{n}$. $x_{vi}$ denotes the value of the *v*-th indicator corresponding to the *i*-th sample, and *n* denotes the number of samples.

**Figure 5:**
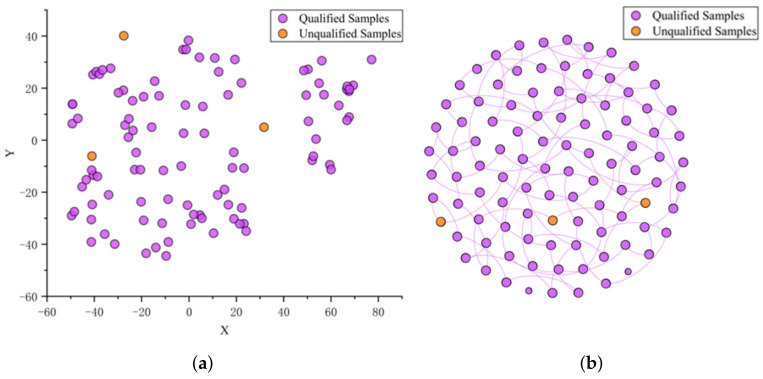
Effect of preprocessing and structuring operations on the original detection data (t-SNE visualization with 100 randomly selected samples). Subfigure (**a**) shows the dimensionality reduction distribution of the sterilized milk detection data before preprocessing. Subfigure (**b**) shows the result of preprocessing and structuring the original data with node diameters proportional to PageRank scores, coloring the node according to sample classes and the edge colors determined by the source node colors. (**a**) Before data preprocessing. (**b**) After data preprocessing and structuring.

We construct a representation based on the correlations between detection samples. Expressly, we represent the food detection samples as nodes in the graph. The testing indicators of the samples are represented as node attributes in the graph. Accordingly, the construction of the attribute graph is completed. The distances between each sample are calculated separately and arranged in descending order when constructing the sterilized milk detection data graph. Our experiments show that the model performs best comprehensively when the top *Z* = 50 samples with the closest distance are set as edged, and the rest are set as unedged. CSGNN is based on self-supervised learning to detect and analyze all samples in a dataset. It uses preprocessing and structuring operations to process the original complex and discrete detection data into the structured representation suitable for GNN. To demonstrate the impact of preprocessing and structuring operations on the initial detection data, we visualized 100 samples (randomly selected) as an example. Figure 5 shows the reduced dimensional distribution of the sterilized milk data before preprocessing and the network structure after preprocessing and structuring, respectively. Our processing is well suited to transform the original detection data between different magnitudes into structured data suitable for GNN without losing as much valid feature information and topological relationships as possible.

#### 3.2.2. Contrastive Instance Pair Sampling

The definition of contrastive instance pairs is the core work of the contrastive learning module. Previous works have demonstrated different advantages in defining instance pairs of graphs [20,43]. Due to the complex topological relationships between different samples of food detection data, we expect the contamination assessment framework for food quality to capture both attribute and structural information of the samples in an integrated manner. This part corresponds to the second part of the content of Figure 3. Inspired by the novel contrastive instance pairs designed for anomaly detection tasks in the paper [21], we focus on modeling the relationships between target nodes and their neighboring subgraphs to help mine the local information of the nodes. Specifically, we use a “sampled samples v.s. adjacent sample groups” instance pair for the attribute network in the CSGNN framework. The first element of the instance pair is a random sample obtained by traversal in the detection data. The second element of the instance pair refers to the group of adjacent samples sampled from the initial samples. The initial sample is set to the sampled sample for positive instance pairs. That means the sampled adjacent sample group matches the nearby samples of the sampled sample. For negative instance pairs, the initial sample is drawn randomly from all samples that do not include the sampled sample; that is, the initial sample will not come from the sampled sample. Therefore, there is a certain degree of mismatch between their sampled samples and adjacent sample groups for contaminated samples. A higher degree of mismatch represents higher hazardous contamination of the detection sample corresponding to that node.

**Figure 6:**
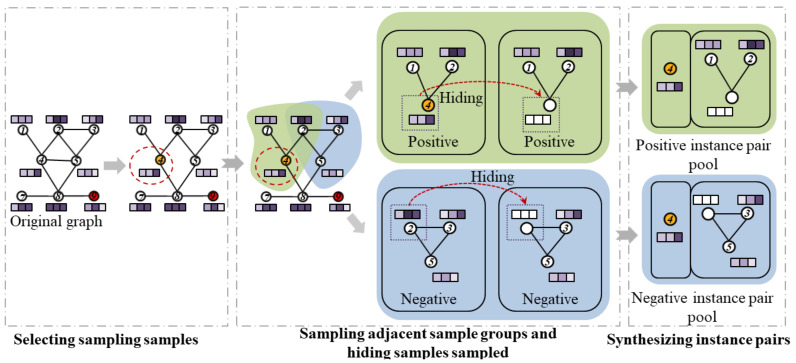
Contrastive learning completes the instance pair sampling process. Node 4 is the sampled sample, and node 9 is the unqualified sample.

Figure 6 shows the sampling process of instance pairs in the CSGNN framework. Sampling consists of selecting the sampled samples, sampling adjacent sample groups, hiding sampled samples, and synthesizing instance pairs.

1. Selecting the sampled samples. All samples in the detection data are traversed randomly within each epoch, and the sampling samples are determined randomly.
2. Sampling adjacent sample groups. We set their initial samples as sampling samples and random sampling samples for the adjacent sample groups of positive and negative instance pairs, respectively. Inspired by the paper [43], we use RWR [44] as the sampling strategy for local sample groups to make the sampling strategy for adjacent sample groups more efficient.
3. Hiding sampled samples. To avoid the contrastive learning module to quickly identify the presence of sampled samples in the adjacent sample groups, we zeroed out the attribute features of the initial samples. That is, the attribute information of the sampled samples is hidden.
4. Synthesizing instance pairs. Combine sampled and adjacent samples into instance pairs and save them to the positive and negative instance pairs sample pool, respectively.

#### 3.2.3. GCN-Based Contrastive Learning

GNNs use information propagation between nodes to capture complex dependencies between data, which has vastly improved the performance of downstream tasks [45,46] such as traffic flow prediction [47], recommender Systems [48], text classification [49], and action recognition [50]. GCNs [51] are multilayer graph convolutional networks that perform first-order local approximations to spectral graph convolutions neural networks, which solves the problem of its inability to maintain translation invariance on discrete non-Euclidean data while preserving the CNN’s ability to process spatial features efficiently. As the third part of Figure 3 demonstrates, in the CSGNN framework, we select the GCN as the backbone of the GNN module, which is an essential component of the CSGNN framework. The sampled instance pairs are used to train the GCN-based contrastive learning model. We perform operations on each batch of instance pairs $I_{i}=\left(s_{i},G_{i},y_{i}\right)$, where $s_{i}$ denote the sampled samples in the instance pair, $G_{i}$ denotes the group of adjacent samples in the instance pair, and $y_{i}$ denotes the labels of the sampled samples. The GCN-based contrastive learning model mainly consists of a GCN module, a dimensionality reduction module, and an embedding comparison recognition module.

GCN module. This module efficiently mines the feature information of the sampled sample $s_{i}$ and the adjacent sample group $G_{i}$, mapping the embeddings of these two parts to the same embedding space, which will prepare the features for comparison between these two parts later. The layer-by-layer propagation principle of GCN for the adjacent sample group is shown in Equation (4).

(4) $$H_{i}^{\left(\ell +1\right)}=\phi \left({\tilde{D}}_{i}^{-\frac{1}{2}}{\tilde{A}}_{i}{\tilde{D}}_{i}^{-\frac{1}{2}}H_{i}^{\left(\ell \right)}W^{\left(\ell \right)}\right)$$

Here, $H_{i}^{\left(\ell \right)}$ refers to the representation matrix learned by the *ℓ*-th implicit layer, the input $H_{i}^{\left(0\right)}=X_{i}$, where $X_{i}$ is the matrix of attribute vectors, and the output is labeled as the embedding $E_{i}$ of the adjacent sample group $G_{i}$. ${\tilde{D}}_{i}$ refers to the degree matrix of the adjacent sample group $G_{i}$. ${\tilde{A}}_{i}=A_{i}+I$ refers to the adjacency matrix with self-connected subgraph, where I refers to the identity matrix. $W^{\left(\ell \right)}\in R^{d^{\left(\ell \right)}\times d^{\left(\ell +1\right)}}$ refers to the *ℓ*-th layer trainable weight matrix. ϕ(·) denotes the activation function such as ReLU.

Compared with the adjacent sample group $G_{i}$, the sampled sample $s_{i}$ has no structural information, so we only need to use the weight matrix of GCN and the corresponding activation function to complete its attribute information feature conversion, as shown in Equation (5).

(5) $$z_{i}^{\left(\ell +1\right)}=\sigma \left(z_{i}^{\left(\ell \right)}W^{\left(\ell \right)}\right)$$

where $z_{i}^{\left(\ell \right)}$ is the row vector of feature representations of the sampled sample $s_{i}$ learned by the *ℓ*-th implicit layer. $W^{\left(\ell \right)}$ is the weight matrix shared with the GCN. The input $z_{i}^{\left(0\right)}$ is defined as the row vector of attributes of the sampled sample $s_{i}$, and the output is labeled as the embedding $e_{i}^{ss}$ of the sampled sample $s_{i}$.

Embedding comparison recognition module. This module maps the high-dimensional sample embedding $E_{i}$ in the adjacent sample group $G_{i}$ to the low-dimensional embedding space, facilitating comparison with the low-dimensional embedding feature $e_{i}^{ss}$ of the sampled samples. The principle is shown in Equation (6).

(6) $$e_{i}^{as}={Dim}^{reduction}\left(E_{i}\right)=\sum_{m=1}^{n_{i}}\frac{{\left(E_{i}\right)}_{m}}{n_{i}}$$

where ${\left(E_{i}\right)}_{m}$ refers to the *m*-th rows of the adjacent sample group embedded in $E_{i}$, and $n_{i}$ refers to the sample size in the adjacent sample group $G_{i}$.

Dimensionality reduction module. This module completes the embedding comparison of sampled samples and adjacent sample groups and is a vital part of the GNN-based contrastive learning model. Inspired by the literature [40], we apply a simple bilinear scoring function to this module. Equation (7) shows the details.

(7) $$Comparator\left(e_{i}^{as},e_{i}^{ss}\right)=\sigma \left(e_{i}^{as}W^{\left(b\right)}e_{i}^{ssT}\right)$$

where $W^{\left(b\right)}$ refers to the weight matrix of the comparison recognition module and ϕ(·) denotes the sigmoid function.

Loss function. We use the standard binary cross-entropy (BCE) loss, applied to contrastive self-supervised learning tasks [20]. Unlike the BCE applied to category balancing as elaborated in the original paper, to effectively deal with the problem of category imbalance in food testing tasks, we perform balanced sampling between positive and negative instance pairs. Therefore, we use a common BCE. We follow Equation (8) to calculate separately the total The prediction score $p_{i}$ for each instance pair $I_{i}=\left(s_{i},G_{i},y_{i}\right)$ for each batch with batch number M. The loss function operation performed on $I_{i}$ is shown in Equation (9).

(8) $$p_{i}=CLM\left(s_{i},G_{i}\right)$$

(9) $$L=-\sum_{i=1}^{M}y_{i}\log\left(p_{i}\right)+\left(1-y_{i}\right)\log\left(1-p_{i}\right)$$

Here CLM(·) denotes the contrastive learning model.

#### 3.2.4. Contamination Assessment

This part corresponds to the fourth part of Figure 3. After the GNN-based contrastive learning is completed, We assess the corresponding sample’s hazardous contamination by identifying the consistency between the sampled sample $s_{i}$ and the adjacent sample group $G_{i}$. Ideally, the lower a sample’s hazardous contamination, the closer the prediction score of its positive and negative instance pairs is to the median value (0.5). Conversely, the higher the hazardous contamination of a sample, the closer the prediction scores of its positive and negative instance pairs are to either side of 0 or 1. We define the contamination value of a sample as the absolute value of the difference between positive and negative instance pairs. Considering the incompleteness and chance of adjacent sample group $G_{i}$ selection, we use multiple rounds of sampling to sample the testing samples. Specifically, we sample each sample in the detection data and sample the positive and negative instance pairs using the sampling strategy introduced in Section 3.2.2. The sampled instance pairs $I_{i}$ are fed into the contrastive learning model, and their prediction scores $p_{i}$ are calculated separately according to Equation (8).

Finally, the sample $f\left(s_{i}\right)$ value-at-contamination is calculated by averaging the absolute values of the prediction scores between positive and negative pairs of instances by subtraction over multiple rounds of sampling, as shown in Equation (10).

(10) $$f\left(s_{i}\right)=\frac{\left|\sum_{r=1}^{R}\left(p_{i,r}^{\left(-\right)}-p_{i,r}^{\left(+\right)}\right)\right|}{R}$$

where *R* is the number of sampling rounds. f(·) is the mapping function of the detected data contamination values, which is the final objective function of the CSGNN framework.

### 3.3. Evaluation Metrics

The objective of the CSGNN framework in the contamination assessment process is to detect unqualified samples and complete the contamination hierarchy for qualified samples. We selected the following five evaluation metrics based on this objective.

The area under the ROC curve AUC will combine the ability of the model to detect qualified and unqualified samples and reasonably assess the comprehensive performance of the CSGNN framework in a dataset with category imbalance. Recall of unqualified samples, which reflects the probability that an unqualified sample is mistakenly detected as a qualified sample, is used to evaluate the model’s ability to detect all unqualified samples. Precision reflects the probability that all detected unqualified samples are actually unqualified samples. We will use precision (all samples) and precision of qualified samples to measure the model’s ability to identify unqualified samples and qualified samples, respectively. The false acceptance rate (FAR), which reflects the probability that the qualified sample is detected as an unqualified sample, is used to measure the model’s contamination warning ability for qualified samples.

The meanings of the four base indicators TP, FP, FN, and TN in the confusion matrix are shown in Table 5. We mark the unqualified samples in the dataset as 1 and the qualified samples as 0. The evaluation metrics AUC, precision, the precision of qualified samples, recall of unqualified samples, and FAR are calculated in Equations (11)–(15), respectively.

(11) $$AUC=\frac{\sum I(\;\mathrm{test}\;\mathrm{qualified}\;>\;\mathrm{test}\;\mathrm{unqualfied}\;)}{\left(TP+FN)*(FP+TN\right)}$$

(12) $$\begin{array}{ccc} Precision & =\frac{TP}{TP+FP} & =\frac{\;\mathrm{The}\;\mathrm{number}\;\mathrm{of}\;\mathrm{unqualified}\;\mathrm{samples}\;\mathrm{correctly}\;\mathrm{tested}}{\;\mathrm{The}\;\mathrm{number}\;\mathrm{of}\;\mathrm{samples}\;\mathrm{tested}\;\mathrm{as}\;\mathrm{unqualified}\;} \end{array}$$

(13) $$\begin{array}{ccc} Precision_{\mathrm{qualified}\;} & =\frac{TN}{TN+FN} & =\frac{\mathrm{The}\;\mathrm{number}\;\mathrm{of}\;\mathrm{qualified}\;\mathrm{samples}\;\mathrm{correctly}\;\mathrm{tested}\;}{\mathrm{The}\;\mathrm{number}\;\mathrm{of}\;\mathrm{samples}\;\mathrm{tested}\;\mathrm{as}\;\mathrm{qualified}\;} \end{array}$$

(14) $$\begin{array}{ccc} Recall_{\mathrm{unqualified}\;} & =\frac{TP}{TP+FN} & =\frac{\mathrm{The}\;\mathrm{number}\;\mathrm{of}\;\mathrm{unqualified}\;\mathrm{samples}\;\mathrm{correctly}\;\mathrm{tested}\;}{\;\mathrm{The}\;\mathrm{number}\;\mathrm{of}\;\mathrm{unqualified}\;\mathrm{samples}\;} \end{array}$$

(15) $$\begin{array}{ccc} FAR & =\frac{FP}{FP+TN} & =\frac{\mathrm{The}\;\mathrm{number}\;\mathrm{of}\;\mathrm{falsely}\;\mathrm{tested}\;\mathrm{as}\;\mathrm{unqualified}\;}{\;\mathrm{Total}\;\mathrm{number}\;\mathrm{qualified}\;\mathrm{samples}\;} \end{array}$$

### 3.4. Baseline Model

We selected three supervised models and two unsupervised models as the baseline model. The supervised baseline model is chosen as NNLM, CNN, and GCN, and the unsupervised baseline model is chosen as LOF and GAN. It is worth noting that the baseline model uses the same data preprocessing as CSGNN and the three supervised models use the same dataset partitioning to ensure the fairness of the comparison.

#### 3.4.1. NNLM

NNLM is a classic shallow neural network model in natural language processing, which proposes the introduction of word vectors in the model for the first time, and successfully breaks through the limitations of the N-gram model in modeling the relationship between words and words. NNLM is an excellent way to learn complex relationships between words and has played a role in the detection task [52], so it is used as the first baseline model in this paper. We set the number of hidden layer neurons to 16, the learning rate to 0.00001, the batch size to 16, and the epoch to 30.

#### 3.4.2. CNN

CNN’s expertise in capturing local feature information in data is widely used in image recognition and speech recognition. It has recently been applied to biometrics and food safety tasks with significant results [53]. We will use CNN as the second baseline model of this paper, setting two different convolutional kernels with four each, the activation function as ReLU, the optimizer as Adam, the learning rate as 0.001, the batch size as 32, and the epoch as 20.

#### 3.4.3. GCN

GCN can simultaneously mine the attribute and structural information in the topology diagram for end-to-end learning, the mainstream GNN model. GCN has more robust feature extraction capabilities than CNNs, solving the problem that CNNs cannot maintain panning invariant in non-Euclidean data, and can effectively mine complex correlation information in data, showing promising potential in classification tasks [54]. Therefore, we will use GCN as the third baseline model in this paper to explore the effect of the GNN algorithm in the food detection task, setting up two convolutional layers, the activation function as ReLU, the optimizer as Adam, with the learning rate of 0.01 and the epoch of 200.

#### 3.4.4. LOF

LOF is a density-based unsupervised anomaly detection algorithm that determines whether a data point is abnormal by comparing the local neighbor density of each data point with its neighborhood data point [55]. Inspired by anomaly detection in attribute networks, we found that anomaly detection has a similar principle to food detection tasks, with the goal of anomalous mining data with feature information that differs from most of the data. Therefore, we will use LOF as the fourth baseline model for this paper to explore the effect of this class of anomaly detection algorithms in food detection tasks.

#### 3.4.5. GAN

GAN comprises the generator (G) and discriminator (D), a generative model based on unsupervised learning methods widely used in image generation and style migration. It also exhibited good performance in the detection and classification tasks [56], so this paper used GAN as the fifth baseline model. We chose Adam as the optimizer for G and D, ReLU as the activation function, the learning rate is 0.0001, the batch size is 32, and the epoch is 500.

### 3.5. Parameter Settings

In the structured representation phase of the data, we construct network G in the same way as the GCN model in the baseline model. During the contrastive instance pair sampling phase, we set the size of the instance pair to $G_{i}$ of the adjacent sample group in the $I_{i}$ to 5. The available nodes will be reused to reach the set size for nodes smaller than the adjacent sample group $G_{i}$ fixed size. In the GCN-based contrastive learning phase, set the number of module layers to 1 and the embedded dimension fixed to 6. We chose Adam as the optimizer. In addition, Set the learning rate to 0.006, the batch size to 450, and the epoch to 1000. We set the sampling round *R* to 256.

## 4. Experiments and Analysis of Results

In this section, we conducted a complete experiment and gave a detailed experimental comparison and analysis. We explain the entire experimental validity by the following three main questions.

Q1: What are the advantages of the CSGNN framework over the baseline model? What performance will these advantages demonstrate in real-world application scenarios?

Q2: What kind of contamination warning does the CSGNN framework enable in contamination assessment applications for food quality? How does it do it?

Q3: Is there a reasonable and feasible explanation for the contamination classification of the CSGNN framework in contamination assessment for food quality?

We will answer each of these questions in the following content and elaborate on the details of our experiment.

### 4.1. Analysis of Results

#### 4.1.1. For Q1 (What Are the Advantages of the CSGNN Framework over the Baseline Model? What Performance Will These Advantages Demonstrate in Real-World Application Scenarios?)

We completed a comparison experiment between five baselines and CSGNN models on the sterilized dairy product detection data, as shown in Table 6. Overall, the CSGNN model performed better than all baseline models. Specifically, we have the following findings.

Firstly, in all models, the AUC values of GCN, LOF, and CSGNN were higher than 0.91. The AUC value of GCN is the highest value of 0.9988 of all models, indicating that the GNN algorithm exhibits very stable performance in food detection tasks. For the AUC values in unsupervised models, the LOF model performed best at 0.9150, possibly because the goal of the anomaly detection task was to find a small number of anomalies in most of the data, and its purpose was to target categorical imbalances. The CSGNN is second only to the LOF model 0.001, indicating that the GCN-based contrastive learning module in the CSGNN framework can show stable performance when dealing with categorical imbalance data, which is critical in practical application scenarios.

Secondly, all models have precision values above 0.95. However, the precision values for GCN and CSGNN are the highest in supervised and unsupervised models. For supervised models, the precision value of GCN 0.9979 is 0.0146 higher than that of the sub-optimal model CNN. For the unsupervised model, the precision value of CSGNN is 0.9829 higher than that of the sub-optimal model LOF. In addition, both GCN and CSGNN had a precision of qualified sample value of 1.0000, which was 0.0096 more heightened than the sub-optimal model NNLM in all models. This shows that the GNN algorithm can better identify qualified and unqualified samples by mining complex correlation information in the detection data, showing promising potential in food detection tasks.

Thirdly, the recall of the unqualified sample value of both GCN and CSGNN is 1.0000, which is more than 13% higher than the sub-optimal model LOF in all models. The recall of unqualified samples reflects the model’s ability to check the unqualified samples in the detection data, which is the essential task for contamination assessment of food quality. For other baseline models, the reason they cannot successfully detect all the unqualified samples may be a bottleneck in mining complex feature information between samples of detection data. The two GNN models can accurately detect all unqualified samples, indicating that the GNN algorithm has successfully captured the detection data’s attribute information and topology information in the food detection task and has good application potential. Further, the CSGNN framework provides a solution for GNN algorithms in unsupervised applications.

Fourthly, the contamination warning task for food quality aims to mine unqualified and potentially contaminated qualified samples in the detection data. To smoothly identify potentially contaminated qualified samples, the FAR value in the food detection task is not as low as possible because the FAR value that is too low will not be conducive to our contamination classification of qualified samples. For the FAR value of the supervised model, NNLM is more suitable for the contamination classification task of detection data. The performance of GCN and CNN is too low and high, respectively, which is not conducive to good contamination classification. For the FAR values of the unsupervised model, LOF and GAN are too low and too high, respectively, and are unsuitable for contamination classification. CSGNN can better achieve the contamination classification of detection data because the CSGNN framework effectively solves the problem that GNN is too low in this metric by setting hyperparameters. The specific scheme is detailed in Section 4.1.2.

#### 4.1.2. For Q2 (What Kind of Contamination Warning Does the CSGNN Framework Enable in Contamination Assessment Applications for Food Quality? How Does It Do It?)

Specifically, the CSGNN framework is divided into three stages: preprocessing and composition, training learning, and contamination assessment. In the preprocessing and composition stage, the unitless processing of the detection data is realized, and the structured representation is completed. During the training learning stage, the contrastive learning model completes the training of the instance pair in a self-supervised manner. In the contamination assessment stage, the contamination value of each sample is obtained, and both the detection data’s binomial classification and contamination level classification is completed accordingly.

We define the contamination value of each sample as the absolute value of its prediction score subtracted between positive and negative instance pairs. In the experiment, we labeled the lowest value of the contamination value of the unqualified samples in the dataset as *W*. *W*, to some extent, reflects the boundary value of the contamination value of the samples with higher contamination probability in the dataset, which is the reason why we set it as the threshold value in the process of dichotomous determination between qualified and unqualified samples by the model for the detection data. Ideally, the greater the hazardous contamination of the contaminated samples after pretreatment, the more the prediction scores of positive and negative instance pairs are distributed to both sides of 0 and 1. The prediction scores of positive and negative instance pairs are close to the qualified samples’ median value (0.5). Accordingly, the contamination value of *U* = 0.5 can be used as a conservative boundary value to distinguish the contaminated samples from the negligible contamination samples by default. We classify the testing samples into four contamination classes based on the contamination value: negligible, low, medium, and high. Each contamination level is classified based on the following.

• Negligible contamination: negligible contamination level, samples are basically risk-free. 0 ⩽ contamination value <*U* for qualified samples.
• Low contamination: Low contamination level, samples with a low probability of being at risk. *U*⩽ contamination value <*W* for qualified samples.
• Medium contamination: Medium contamination level, samples with a high probability of being at risk. *W*⩽ contamination value ⩽ 1 for qualified samples.
• High contamination: High contamination level, all unqualified samples belong to this category.

**Figure 7:**
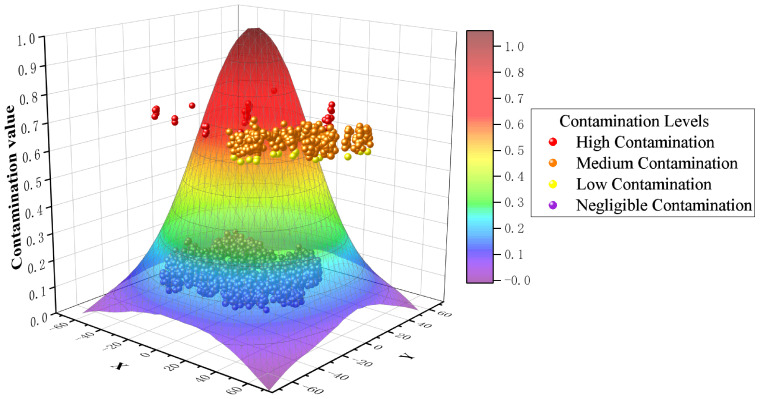
Contamination ranking of the CSGNN framework on sterilized dairy products detection dataset.

To observe the contamination warning effect of the CSGNN framework more intuitively, we made a visual display, as shown in Figure 7. Based on the distribution of contamination values of the samples, they are sequentially classified as negligible contamination samples, low contamination samples, medium contamination samples, and high contamination samples.

#### 4.1.3. For Q3 (Is There a Reasonable and Feasible Explanation for the Contamination Classification of the CSGNN Framework in Contamination Assessment for Food Quality?)

**Figure 8:**
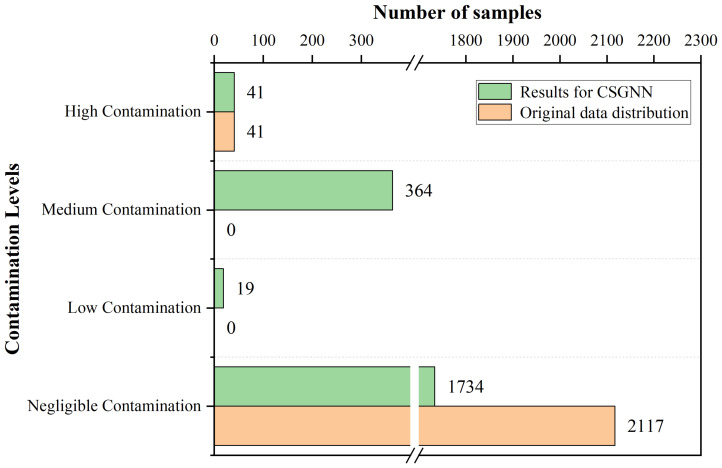
Division of sample numbers for each contamination level of the CSGNN framework on the sterilized dairy products detection dataset.

**Figure 9:**
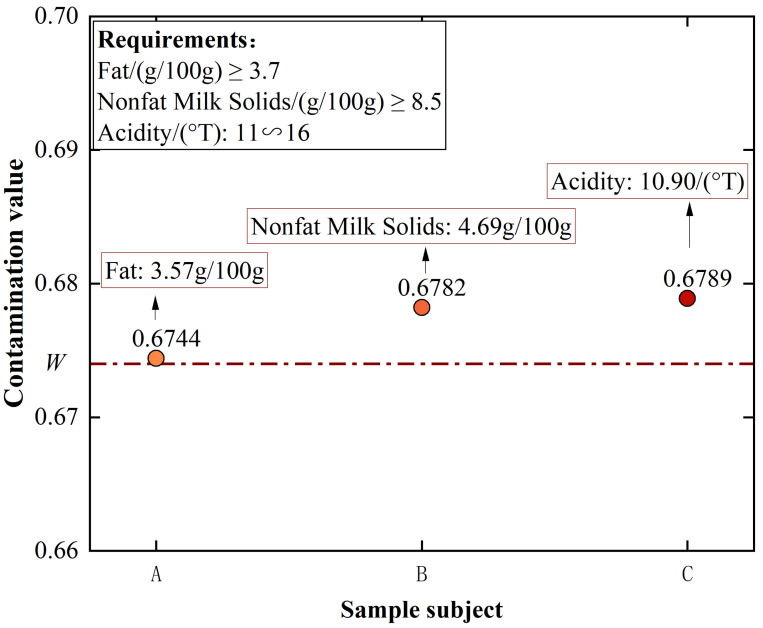
The specifics of three unqualified sample objects whose contamination values from the CSGNN framework are closest to the *W* values.

From Figure 7, it can be found that the same contamination level samples exist in clusters in the graph with a minimal distance between samples. This is because the difference between the contamination values of samples with the same contamination level is minimal. At the same time, there is a significant difference between the contamination values of samples with different contamination levels. No sample does not comply with the principle, which indicates that our principle of dividing samples with varying levels of contamination is feasible. Specifically, for the 2158 sterilized milk detection data, 1734 of the 2117 qualified samples had contamination values below 0.17 and were classified as negligible contamination by the CSGNN framework. 41 unqualified samples had a minimum contamination value of W=0.674 and were all classified as high contamination. 19 qualified samples had contamination values above 0.17, ranked in the low contamination sample class. The 364 qualified samples with contamination values between *W* and 1 were classified as medium contamination samples. As shown in Figure 8, The CSGNN framework successfully detects all unqualified samples from the original sterilized milk detection data while classifying the qualified samples specifically classified into three levels according to the contamination value: medium contamination, low contamination and negligible contamination. To further observe the detection performance of the CSGNN, As shown in Figure 9, we checked the contamination values of the three nearest unqualified samples A, B, and C, which was 0.6744, 0.6782, and 0.6789, respectively, for *W*. We further examined the specific values of the six evaluation metrics for these three samples, where sample A unqualified due to its fat content of 3.57, which was below the standard range minimum value of 3.7, and sample B was unqualified because its nonfat milk solids content was 4.69, which was lower than the minimum value of 8.5 in the standard range. Sample C was unqualified because its acidity content was 10.90, which did not meet the standard range of 11∼16.

In addition, for the contamination values of negligible contamination and contaminated samples in the dataset, the CSGNN shows a significant difference (A cliff-like gap between the contamination values of negligible contamination and contaminated samples), and the boundary value distinguishing the two is much lower than the default *U* value, even only 0.17. our framework highlights the information difference between the negligible contamination and contaminated samples in the preprocessing process. The chance judgment caused by local perception is smoothly avoided in the contrastive learning process of multiple rounds of sampled instance pairs. The prediction scores of the instance pairs corresponding to the negligible contamination samples are realized to be infinitely close to the median value (0.5) in calculating the contamination value. In contrast, the prediction scores of the instance pairs corresponding to the contaminated samples are distributed to both sides of 0 and 1. In summary, the CSGNN framework has a significant hierarchy of contamination values for sterilized dairy product detection samples and efficiently achieves a reasonable classification of food safety contamination levels.

The CSGNN framework performs data preprocessing and optimal composition for unknown food detection data. The data are fed into a feedforward neural network to obtain the contamination value of each testing sample. This process focuses on the lowest contamination value *W* of the unqualified samples and the more obvious boundary value *U* between the contaminated and negligible contamination samples. (Considering each detection data’s variable quality, we will default *U* = 0.5 if no obvious *U* value is observed). The framework will achieve the contamination classification of different food detection data based on the *W* and *U* values obtained from different data according to the principles described in Section 4.1.2.

### 4.2. Application and Optimization of the CSGNN

Inspired by [57] and considering the rigor of government work, we introduce a panel of experts from food quality supervision departments to participate in the example analysis and application. The CSGNN framework can provide technical support and the theoretical basis for implementing food safety supervision and effectively improve the efficiency of food quality contamination warnings by the expert team in the detection of massive, diverse types and complex relationships of food data in practical application scenarios. The expert team analyzes and implements more refined regulatory decisions based on CSGNN’s quality contamination warning results, ensuring the reasonableness and stability of the safety and quality assessment results.

Our intelligent food safety supervision platform, built in cooperation with the Institute of Product Quality Supervision and Inspection in a Chinese province, promotes the application of CSGNN on the ground. We encapsulated the CSGNN framework to provide technical support for this platform’s hazard contamination warning module. For a batch of detection data of any food type, CSGNN takes the detection data of *N* samples *V* detection indicators $x_{v1,\ldots ,}x_{vn}$ as input (as shown in the “Item” column in Table 3), and the output is the contamination level of *N* samples. Our framework shows good detection performance during online testing of the input data, and feeds the results to the terminal according to the contamination level. The regulatory staff analyzes the results and takes appropriate warning measures to reduce the adverse effects of hazardous contaminated products.

## 5. Discussion

CSGNN provides an efficient end-to-end approach for contamination warning and control of food quality based on self-supervised learning, which can generate significant social and economic benefits while consuming lower costs. Specifically, the CSGNN model can quickly perform food quality detection on an ordinary computer, requiring minimal financial and time costs and no human intervention. At the same time, the model can effectively optimize the food safety and quality control system in an early warning way, and reduce the economic loss (financial penalty) and sales loss due to the damage to the company’s reputation caused by unqualified products. For example, a food safety incident occurred in 2022 in which propylene glycol was detected in pure milk produced by Maiquer Group (https://www.samr.gov.cn/xw/zj/202207/t20220703_348326.html accessed date: 15 February 2023), which not only caused the company to be fined tens of millions of dollars (https://static.cninfo.com.cn/finalpage/2022-08-23/1214356756.PDF accessed date: 15 February 2023). More importantly, it seriously affected the brand’s trust in consumers’ minds and caused irreparable cascading negative impacts on the company’s business performance.

Combined with the analysis of the results of the contamination classification provided by CSGNN, food safety regulators will establish an emergency mechanism to deal with special hazards. Specifically, recall of unqualified samples from food detection data and further traceability of testing indicators that lead to samples with hazard contamination. Thus, a priority system for hazard analysis and effective measures for safety regulation will be established, and unified decisions on profit and loss of economic and food safety will be taken from a global perspective. Hazard contamination of food is closely related to food producers, and the theoretical basis provided by the CSGNN detection results will help regulators to establish food safety control strategies based on scientific principles and strengthen the effectiveness of regulation of food producers. Thus, they are urged to focus on the content of testing indicators that often lead to samples with hazardous contamination in the production process. This can optimize the preventive principle of hazard contamination in food production and processing, and efficiently supervise and guide food producers’ benign production to minimize the occurrence of food hazard contamination accidents.

## 6. Conclusions and Future Work

This paper proposes applying GNN-based contrastive self-supervised learning to contamination warning and control of food quality. This is the first attempt at GNN-based self-supervised learning in food safety early warning analysis. We innovatively proposed an end-to-end contamination assessment framework for food quality called CSGNN. The framework directly models the complex feature associations between the detection data. It uses contrastive self-supervised learning to construct positive and negative instance pairs to train the detection data of category imbalance. Finally realizes the detection of unqualified samples and the contamination level of qualified samples by obtaining the contamination value of each detection sample. We applied the framework to a batch of sterilized milk detection data in a province in China, and its recall of unqualified samples and AUC values reached 1.0000 and 0.9188, respectively, which indicates that our framework can detect all unqualified samples and show better stability and lower false detection rate in the practical application of data category imbalance. Experimental results show that the CSGNN framework successfully mines the attribute information and structural information between different indicators of food detection data, and the final contamination value of the detection sample reflects its contamination level well to achieve efficiently. Our research provides new ideas for contamination assessment of food quality, and food safety regulators can make more efficient decisions based on CSGNN detection results combined with expert panels.

There is a degree of subjective interference in the parameters of CSGNN in contamination classification (which is set to 0.5 by default when a more conservative boundary value *U* between contaminated and negligible contamination samples is not observable in the detection data). In future work, we will further explore using a parameter-free approach to risk ranking to ensure the objectivity of the process, e.g., data analysis of contamination values of samples utilizing mathematical statistics to determine the contamination rank of each sample. In addition, we will more fully consider information on food hazard contamination and toxicology to optimize the contamination assessment model for food quality in subsequent studies.

## Figures and Tables

**Table 1:** Notes and explanations related to the CSGNN framework. The table’s three blocks (from top to bottom) show the data preprocessing and structured representation, GCN-based contrastive learning, and CSGNN’s hyperparameters, respectively.

Notation	Description and Explanation
$x_{v}^{\min}$	The minimum value of the original value of the *v*-th indicator for all samples.
$x_{v}^{\max}$	The maximum value of the original value of the *v*-th indicator for all samples.
$x_{v}^{\mathrm{mean}}$	The average value of the original value of the *v*-th indicator for all samples.
$x_{vn}$	The original value of the *v*-th indicator of the *n*-th sample.
*N*	The number of samples in the detection data.
*V*	The number of testing indicators in the detection data.
*n*	The number of nodes in G.
*d*	The dimension of the attribute in G.
G=(V,E,X)	Attribute networks constructed from the detection data.
V	The set of nodes of G.
E	The set of edges of G.
$X\in R^{n\times d}$	The attribute matrix of G.
$I_{i}=\left(s_{i},G_{i},y_{i}\right)$	Instance pairs for each batch with a total batch size of *M*.
$s_{i}$	Sampled samples in instance pair $I_{i}$.
$G_{i}$	The group of adjacent samples in instance pair $I_{i}$.
$y_{i}\in \left\{0,1\right\}$	True label of the sampled sample $s_{i}$.
$H_{i}^{\left(\ell \right)}\in R^{a\times b^{\left(\ell \right)}}$	The representation matrix is learned by the *ℓ*-th implicit layer.
$W^{\left(\ell \right)}\in R^{d^{\left(\ell \right)}\times d^{\left(\ell +1\right)}}$	The *ℓ*-th layer trainable weight matrix.
$E_{i}\in R^{a\times b}$	The embedding matrix of the nodes in $G_{i}$.
$z_{i}^{\left(\ell \right)}\in R^{b^{\left(\ell \right)}}$	The row vector of feature representations of the sampled sample $s_{i}$ learned by the *ℓ*-th implicit layer.
$e_{i}^{ss}\in R^{b}$	The embedding vector of $s_{i}$.
$e_{i}^{as}\in R^{b}$	The embedding vector of $G_{i}$.
$W^{\left(b\right)}\in R^{b\times b}$	The weight matrix of the comparison recognition module.
$p_{i}$	The prediction score of $I_{i}$.
$f\left(s_{i}\right)\in \left[0,1\right]$	The contamination value of the sampled sample $s_{i}$.
*R*	The number of sampling rounds.
*a*	The number of nodes in adjacent sample groups.
*b*	The dimensionality of embedding.
*W*	The lowest contamination value of the unqualified samples.
*U*	The more obvious boundary value between the contaminated sample and the negligible contamination sample (default 0.5).
*Z*	Set the number of edges when structuring.

**Table 2:** The specific requirements of the six testing indicators and testing methods ${}^{1}$.

Item		Requirements	Testing Method
physicochemical index	Lactose/(g/100 g)	⩽2.0	GB 5009.8-2016
	Protein/(g/100 g)	⩾3.1	GB 5009.5-2010
	Acidity/(°T)	11∼16	GB 5413.34-2010
	Fat/(g/100 g)	⩾3.7	GB 5413.3-2010
	Nonfat Milk Solids/(g/100 g)	⩾8.5	GB 5413.39-2010
mycotoxin index	Aflatoxin M${}_{1}$/(μg/kg)	⩽0.5	GB 2761-2017

^1^http://down.foodmate.net/standard/index.html (accessed date: 10 August 2022)

**Table 3:** Detection data for partially sterilized dairy products from 2013 to 2021.

Sample Number	Testing Date	Item					
		Lactose	Nonfat Milk Solids	Protein	Acidity	Aflatoxin M${}_{1}$	Fat
20211010-578	10 October 2021	1.73	8.97	3.40	12.00	0.2	4.28
20200410-525	10 April 2020	1.74	8.67	3.39	12.08	0.5	4.69
20190504-166	4 May 2019	1.72	8.81	3.37	12.07	0.5	4.44
20180610-453	10 June 2018	1.70	8.68	3.25	12.19	0.5	4.36
20210909-512	9 September 2021	1.73	8.62	3.42	12.40	0.2	4.20

**Table 4:** Sterilized dairy products testing indicators categories classification.

Categories	Item	Requirements
Positive indicators	Aflatoxin M${}_{1}$/(μg/kg)	⩽0.5
	Lactose/(g/100 g)	⩽2.0
Inverse indicators	Protein/(g/100 g)	⩾3.1
	Fat/(g/100 g)	⩾3.7
	Nonfat Milk Solids/(g/100 g)	⩾8.5
Oscillatory indicators	Acidity/(°T)	11∼16

**Table 5:** Meaning of base indicators in confusion matrix.

		Real Label	
		1	0
Predicted Label	1	True Positive (TP)	False Positive (FP)
	0	False Negative (FN)	True Negative (TN)

**Table 6:** All models were evaluated 30 times on the sterilized milk detection dataset and averaged the results. (In input, X indicates the input data information, and Y represents the Label corresponding to the data. The best performance values of each evaluation metric in the supervised and unsupervised models are shown in bold and bold combined with underlining, respectively. * indicates the best performing model applicable to the food contamination early warning task in each evaluation metric).

Input	Models	AUC	Precision	Precision of Qualified Samples	Recall of Unqualified Samples	FAR
(X,Y)	NNLM	0.7602	0.9668	0.9904	0.7059	0.2941 *
	CNN	0.6765	0.9833	0.9829	0.3529	0.6470
	GCN	**0.9988**	**0.9979**	**1.0000**	**1.0000**	0.0024
(X)	LOF	**0.9150**	0.9787	0.9787	0.8823	0.0523
	GAN	0.5804	0.9546	0.9782	0.2353	0.7647
	CSGNN	0.9140	**0.9829**	**1.0000**	**1.0000**	0.1719 *

## Data Availability

Not applicable.
